# The Principles of Diagnosis

**Published:** 1834-07-01

**Authors:** 


*The Principles of Diagnosis.* By Marshall Hall, m.d., f.r.s. Two Vols. in One.—London, 1834. 8vo. pp. 426.

The self-evident importance of correct diagnosis to the successful treatment of disease renders every attempt at its improvement laudable; but when these attempts, although leaving much to be desired, still achieve nearly all that is possible, there is no praise to which their undertaker is not entitled. In this position we conceive Dr. Marshall Hall has placed himself. Science and humanity are equally his debtors: and long may he enjoy their rewards! This is not unmerited eulogy; and we know that he is superior to the affectation of disclaiming what it has been the labour of his life to deserve.

"One of the sources of diagnosis enumerated constitutes a department of knowledge which may be termed *new:* it is that of the effect of remedies, and especially of bloodletting, as a diagnostic of diseases, and as a criterion of the general powers of the system. In cases in which it is doubtful whether the pain, or other local affection, be the effect of inflammation or of irritation, the question is immediately determined by placing the patient upright, and bleeding to incipient syncope: in inflammation much blood flows, in irritation very little. The violence of the disease, the powers of the system, and the due measure of the remedy, are determined at the same time. *There is, in my opinion, no single fact in physic of equal importance and value in the diagnosis of acute diseases, and the use of an important remedy.*" (P. 3.)

Now, as a general truth, this proposition must meet with universal assent; but the exceptions to it are so striking, that, next in importance to admitting the fact, is insisting upon its occasional fallibility. Every one, and Dr. Marshall Hall especially, must have seen the mischiefs resulting from injudicious depletion; that treatment having been persisted in, in virtue of the apparent impunity with which it was sustained. It will not be contended that bloodletting is always appropriate in erysipelas: indeed, some Brunonians, who are not therefore bad practitioners, never employ it; and, on the contrary, it is a measure never omitted by others. Impartial judges justly condemn both these extremes; for it hardly ever fails to afford temporary relief, and rarely furnishes the evidence of Dr. Hall's test at the time of its application. No; the evil is manifested in precipitating the typhoid termination of severe erysipelas. Therefore, the man whose bloodletting in this disease should be regulated by the author's criterion would do incalculable mischief in treating a disease which we believe is oftener one of asthenia than the reverse. Moreover, the same remarks will, with equal justice, apply to rheumatism, in which the present relief afforded by bleeding is in a direct ratio with the permanent mischief; though nothing can exceed the readiness with which the system parts with blood in this disease. In an advanced part of the first volume he recognizes some exceptions to his own rule, but not to the extent which his large experience could supply.

"The progress of medicine as a science—might we not say, as an *abstract* science?—may be considered as greatly dependent on that of our knowledge of morbid anatomy; but the advancement of physic as a practical art is intimately linked with our knowledge of the history, symptoms, and the effects of remedies,—with the diagnosis of the disease in the living patient." (P. 3.)

A more sterling truth was never enunciated, or one more diagnostic of a philosophical physician.

The old notion that sailors are less obnoxious to calculous disorders than landsmen is perpetuated here; and therefore we infer that Dr. Hall does not accept the plausible explanation that the two extremes of existence, infancy and old age, furnish the largest number of examples of stone in the bladder; and, as both these eras are without the pale of nautical life, the scarcity of the malady among seamen is in good measure accounted for.

"There is a point in the history of diseases which still requires attention, viz. what has been termed the *metastasis*, or *conversion* of diseases. This event has occurred in gout, rheumatism, erysipelas, cynanche parotidea, some cutaneous affections, suppressed hemorrhoids, &c. But I think some of the events which accompany the dyspepsiæ have been mistaken for the *metastasis* of diseases; and some of the effects of the *treatment*, as will be noticed immediately, are very apt to be mistaken for changes or consequences of the disease." (P. 26.)

"In *inflammation of the abdomen, with severe pain*, there is a continued state of contraction of the muscles of the face, inducing an unnatural acuteness of the features; the forehead is wrinkled, and the brows knit; the nostrils are acute, drawn upwards, and moved by the alternate and irregular acts of the respiration; the wrinkles which pass from the nostrils obliquely downwards are deeply marked; the upper lip is drawn upwards,* and the under

* "See Laennec, ed. i., t. i., pp. 90, 398."

one, perhaps, downwards, exposing the teeth; the chin is often marked with dimples. This state of the features is aggravated on any increase of pain, from change of position, muscular effort, or external pressure. Indeed, in cases of abdominal affection, it is better to press on the abdomen, or beg the patient to raise the head and shoulders, and watch the *effect* on the expression of the countenance, whilst the patient's mind is occupied with some other subject, than to ask the direct question whether pressure induces pain, as is usually done; for patients naturally suppose that every *painful* part must also be *tender*, and are therefore apt to answer in the affirmative, although incorrectly," (P. 47.)

While adopting this useful suggestion, we may also remember that a great difference in the effect is produced by the amount of pressure, severity of pain not being always in proportion to that amount. The muscular soreness accompanying some acute affections, sometimes rheumatic—is increased by slight superficial pressure; but if this be persisted in, and converted into a tolerably firm and continued one, the pain diminishes, thus furnishing a diagnostic of the absence of peritoneal inflammation; keeping in mind that in some varieties of erratic childbed, or "peritoneal fevers," excessive tenderness of the abdomen, indeed absolute intolerance of the slightest pressure, has been held not to depend upon inflammation. To establish this valuable fact, no one has contributed more than our author, except perhaps the late Dr. Gooch.

"The presence of morbid changes of structure affords the evidence of previous morbid actions; their absence affords the proof that such morbid actions have not existed." (Vol. i. p. 185.) Surely this last part will not be even generally assented to; for it cannot be denied, nor disproved, that structure and organs shall have been the seat of morbid actions,—ay, and the most intense and disturbing ones, without furnishing any even the slightest post-mortem evidences of their previous existence. To go no further than inflammation, may we not find an entire absence of these indications of disease, yet the patient shall incontestably have suffered their severest forms, stopping short of those structural changes, which the severity of the disease prevented by destroying life before their maturation. Disorders, serious from their intensity and duration, have had their seat in parts whose structure they have failed to disorganize: this is constantly happening, in spite of the dogma that "their absence affords the proof that such morbid actions have not existed."

The following admission is of infinite value, which is enhanced by the fact of its emanating from so high an authority as Dr. Hall, who has a strong partiality for a branch to which he here does entire justice:

"I would observe, however, that the mere student of morbid anatomy is not a good practical physician. There is so much to be considered in the condition of the general system, in the sympathies with the organ, or organs, principally affected, in the effects of remedies, &c. that he who has an eye to the mere disease, the morbid change of structure, alone, is not in possession of the knowledge required to treat the patient." (P. 185.)

Very germane to this matter, but often, very often, forgotten over the victim of the disease, is the following:

"It seems probable, indeed, that the solids, the fluids, and the nervous system, are variously but simultaneously involved in all diseases. The morbid change is seldom confined to a part, an organ. Certain morbid appearances, and certain associations of morbid appearances, are met with in fevers, in the eruptive diseases, in inflammations, in scrofulous or tuberculous affections, in dropsies, in hæmorrhages, &c. to which my attention has been forcibly drawn, and to which I wish to draw the attention of the profession. Such forms and such associations of morbid changes constitute the disease. Each of such forms is peculiar. The same change of structure observed in different diseases, according to our usual phraseology, is not, in fact, the same. The inflammatory affections of the skin, which occur in scarlatina, in rubeola, in variola, are not the same. In like manner morbid changes of structure, observed in febrile, inflammatory, and other diseases, although designated by the same term, are not in truth the same." (P. 6. vol. ii.)

The following observations, too, should never be lost sight of for a moment:

"The investigation into the state of the '*constitution*' of the patient, is one which has been greatly neglected, and which must be studied anew.' It is to this department of knowledge that Celsus alludes in the following paragraph: 'Ob quæ conjicio, eum qui propria non novit, communia tantum intueri debere; eumque qui propria nosse potest, ea quidem non oportere negligere, sed his quoque insistere. Ideoque, cum par scientia sit, utiliorem tamen medicum esse amicum, quam extraneum.' It is to this department of medical knowledge that I would particularly call the renewed attention of the profession. Every physician feels how much easier it is to prescribe for a patient for whom he has frequently prescribed before, than for a stranger. The habit of such a patient in regard to the kind and severity of the disease, and in regard to the power of supporting important remedies, is familiarly known to him. There is in every one a certain idiosyncrasy, to which it is highly important to attend with scrupulous care. This notion is become antiquated of late; it is nevertheless founded in truth, and will meet with acceptance, as an old friend, by all practical physicians." (P. 9. vol. ii.)

"It has been my wish, in the first place, as much as possible, to arrange and bring before the young physician every case which can require his attention in actual practice." (P. 19. vol. ii.)

This, with all deference to the author, we deem unattainable: an accurate calculation is impossible, because many causes are constantly acting as disturbing ones, to prevent any attempt at a nosology of the majority of deviations from healthy function; and these deviations are the diseases on account of which the practitioner is consulted, perhaps, seven times out of ten. He expresses his desire to avoid "surcharging these sketches with the names and descriptions of diseases, which are more objects of curiosity and over-refinement than of practical utility.

"I must be excused for still thinking the terms fever, inflammation, rheumatism, scirrhus, &c., useful and practical designations of disease, just as rubeola, erysipelas, and gout are so." (P. 19, vol. ii.)

However laudable it may be, and we admit that it is so, to supersede arbitrary terms by scientific and descriptive designations, it is to be feared that terms having the force or qualities of definitions, will ever be things to be desired in the study and practice of medicine. Almost equally difficult appears to be what Dr. Hall calls a *diagnostic arrangement* of diseases: a severe critic would encounter but slight difficulty in shewing its fallacy.

We do not think that the reasoning is sound which concludes that, because fifteen hnndred varieties of the rose have been identified, we may therefore expect an approximation to similar success in the diagnostic arrangement of diseases. Comparison and contrast are the elements of the botanical experiment, and proximity of the objects furnishes a ready means of distinguishing identity or difference: far otherwise in disorders, where the amount of resemblance or disparity is ever varying,—where the symptoms cannot be sensibly or tangibly appreciated, as in the case where matter is the thing judged of.

It is not to be expected that fever, the theme of so many pens, should fail to afford Dr. Hall an opportunity of evincing a familiarity with this engrossing and important subject: this he does, and in a way that makes us regret he did not give a large division of his work to the subject.

From the notes of interrogation after the mention of malaria and contagion as the causes of typhus, we are to infer that Dr. Hall doubts those sources being true ones of this fever; a scepticism which he will share with but few, and which the appeal to fact—with him a favourite one, will certainly not fortify. He also appears to regard it always as an idiopathic disease, and one not likely to be superinduced: if we correctly so understand him, we are again dissentient. He denies the inflammation and ulceration of Peyer's glands being the effect, the cause, or the complication of typhus; and affirms that they are an essential part of the disease. Perhaps this point may still be considered as doubtful; but our author's theory is certainly confirmed by the late researches of Dr. Chomel.

We are surprised that, in describing the morbid anatomy of typhus, but slight, if any, notice is taken of the "sticky varnish," well described by Dr. Armstrong as almost invariably lining the bronchial passages; the existence of which may in part account for the condition of the blood, as it undergoes less perfectly the pulmonary changes, in consequence of this peculiar mucous lining preventing the perfect access of the atmosphere.

Admitting the propriety of Dr. Hall's two great divisions of fevers into "continued" and "periodic," his assigning the "remittent forms" a place in the latter is more questionable. In intermittent fevers there is an interval between the recurrence of the symptoms of the disease: in remittent fever there is no interval, but, in lieu of it, a continuity of the disease and symptoms, with a periodic exacerbation of the latter. The diagnostic of remittent fever, the absence of the periodic cold stage, is not alluded to; nor is the state of the pupil dwelt upon as an index to the condition of the brain in fever, independent of effusion.

The chapter on the diagnosis of Irritation, Exhaustion, Delirium Tremens, and Erethismus Mercurialis, is invaluable.

It would be unjust to Dr. M. Hall to withhold the highest praise from his eminently successful labours in a department of his art which he is well known to be practically familiar with, and, consequently, well fitted to write upon. His unwearied zeal in the pursuit of knowledge forbids us to despair that, as he must necessarily advance in the attainment of his favourite objects, we shall at a future period enjoy the fruits of labours even still more useful to mankind.

